# FGF10 Enhances Peripheral Nerve Regeneration *via* the Preactivation of the PI3K/Akt Signaling-Mediated Antioxidant Response

**DOI:** 10.3389/fphar.2019.01224

**Published:** 2019-10-16

**Authors:** Lvpeng Dong, Rui Li, Duohui Li, Beini Wang, Yingfeng Lu, Peifeng Li, Fangzheng Yu, Yonglong Jin, Xiao Ni, Yanqing Wu, Shengnan Yang, Guanxi Lv, Xiaokun Li, Jian Xiao, Jian Wang

**Affiliations:** ^1^Department of Hand Surgery and Peripheral Neurosurgery, The First Affiliated Hospital of Wenzhou Medical University, Wenzhou, China; ^2^Molecular Pharmacology Research Center, School of Pharmaceutical Science, Wenzhou Medical University, Wenzhou, China; ^3^School of Chemistry, Sun Yat-sen University, Guangzhou, China

**Keywords:** fibroblast growth factor 10, axonal regeneration, peripheral nerve injury, oxidative stress, apoptosis

## Abstract

The process of axonal regeneration after peripheral nerve injury (PNI) is slow and mostly incomplete. Previous studies have investigated the neuroprotective effects of fibroblast growth factor 10 (FGF10) against spinal cord injury and cerebral ischemia brain injury. However, the role of FGF10 in peripheral nerve regeneration remains unknown. In this study, we aimed to investigate the underlying therapeutic effects of FGF10 on nerve regeneration and functional recovery after PNI and to explore the associated mechanism. Our results showed that FGF10 administration promoted axonal regeneration and functional recovery after nerve damage. Moreover, exogenous FGF10 treatment also prevented SCs from excessive oxidative stress-induced apoptosis, which was probably related to the activation of phosphatidylinositol-3 kinase/protein kinase B (PI3K/Akt) signaling. The inhibition of the PI3K/Akt pathway by the specific inhibitor LY294002 partially reversed the therapeutic effects of FGF10 both *in vivo* and *in vitro*. Thus, from our perspective, FGF10 may be a promising therapeutic drug for repairing sciatic nerve damage through countering excessive oxidative stress-induced SC apoptosis.

## Introduction

Unlike the central nervous system (CNS), the peripheral nervous system (PNS) retains a certain regeneration capacity, which enables the regrowth of damaged axons and impaired nerves (Chen et al., 2007). Nevertheless, the regeneration rate of peripheral nerves is slow, and proximal nerve segments rarely regrow over long distances to their target organs. As a result, it affects the quality of life of patients and is a major socioeconomic burden (Sulaiman and Gordon, 2009; Gordon, 2016). Because nerve regeneration is a complex process that involves interactions among cellular elements, cytokines, extracellular matrix, and growth factors, the complete recovery of injured nerves is difficult (Caillaud et al., 2018).

Schwann cells (SCs), the myelinating cells of the PNS, are essential for nerve regeneration and for the saltatory conduction of action potentials (Scherer and Wrabetz, 2008). Following nerve injury, denervated SCs begin to dedifferentiate, migrate, proliferate, and transform into regeneration-promoting new cells, called repair SCs (Jessen and Mirsky, 2016). This type of SCs is able to generate a favorable microenvironment for axonal growth by clearing degenerated myelin debris and secreting neurotrophic factors (Jessen et al., 2015). Thus, promoting SCs survival and inhibiting apoptosis are vital for maintaining structural and functional integrity following peripheral nerve injury (PNI).

Oxidative stress, characterized by excessive reactive oxygen species (ROS), is a critical initiating factor for PNI (Yang et al., 2016; Ino and Iino, 2017; He et al., 2018). The overproduction of ROS disturbs the oxidation-antioxidant equilibrium and leads to mitochondrial dysfunction, lipid peroxidation, and cell apoptosis. Furthermore, excessive ROS production in SCs causes dysfunction in DNA synthesis, protein expression, and mitochondrial structure (Finkel and Holbrook, 2000; Wang et al., 2013). Therefore, it is essential to inhibit excessive ROS generation to maintain the SC function and the interactions between SCs and other cell types after PNI.

The nuclear factor transcription erythroid-like factor 2 (Nrf2) plays a critical role in regulating redox homoeostasis. The activation of Nrf2 results in the accumulation of some enzymes, such as heme oxygenase-1 (HO-1), NAD(P)H:quinone oxidoreductase (NQO1), and superoxide dismutase (SOD2) (Son et al., 2013). Nrf2 is also closely related to cell apoptosis through upregulating the expression of Bcl-2, an antiapoptotic protein. Low levels of Bcl-2 expression and increased expression of proapoptotic proteins, including Bax and caspase-3, are typical markers of cell apoptosis (Niture and Jaiswal, 2011; Niture and Jaiswal, 2012; Niture et al., 2014).

The phosphatidylinositol-3 kinase/protein kinase B (PI3K/Akt) pathway has been reported to play a major role in the modulation of axonal growth, myelin sheath formation, and SC function in the PNS. The activation of Akt in SCs increases the expression of myelin proteins, including myelin basic protein (MBP) and myelin basic zero (MPZ), which regulates remyelination. In contrast, inhibiting the PI3K/Akt pathway with LY294002 significantly attenuates SCs migration (Lv et al., 2015; Domenech-Estevez et al., 2016). Furthermore, the inhibition of this signaling pathway also obviously decreases the synthesis of proliferating cell nuclear antigen (PCNA; a marker of cell proliferation) in SCs (He et al., 2011). All of these findings show that the PI3K/Akt pathway modulates the multiple functions of SCs, including migration, proliferation, and remyelination.

FGF10 is a member of the fibroblast growth factors (FGFs) that plays important roles in regulating biological functions such as morphogenesis, proliferation, and the inhibition of apoptosis (Kelleher et al., 2013). FGF10, which was originally identified in rat embryos, mediates biological signaling in a paracrine manner (Itoh, 2016). Furthermore, FGF10 is widely distributed in many organs, such as adipose tissue, the lungs, the limbs, and the prostate, and plays an essential role in regulating cell mitogenesis, proliferation, differentiation, and migration (Min et al., 1998; Thomson and Cunha, 1999; Jimenez and Rampy, 1999; Sakaue et al., 2002). A recent study showed that FGF10 expression in neurons is increased after spinal cord injury (SCI) and that exogenous FGF10 administration induces functional recovery and attenuates the inflammatory response by activating PI3K/Akt signaling in an animal model of SCI (Chen et al., 2017). Another study demonstrated that FGF10 protects neurons and ameliorates cerebral ischemia injury by activating the PI3K/Akt signaling pathway and reducing NF-κB-mediated neuroinflammation (Li et al., 2015b; Li et al., 2016). However, the effects of FGF10 on functional recovery after PNI and the associated molecular mechanism have not been documented to date.

The aim of the present study was to investigate whether FGF10 plays a neuroprotective role in facilitating axonal regeneration and functional recovery after PNI and to explore the related molecular mechanisms. Our results indicate that FGF10 treatment reduces SCs apoptosis, enhances axonal growth and regeneration, and improves functional recovery following PNI. Furthermore, this beneficial effect is most likely regulated by attenuating PI3K/Akt signaling-mediated oxidative stress both *in vivo* and *in vitro*. Collectively, our results suggest that FGF10 performs a certain role and may be a potential agent for the treatment of PNI.

## Materials and Methods

### Reagents and Antibodies

FGF10 was obtained from the School of Pharmacy, Wenzhou Medical University (Wenzhou, China). Antibodies against *p*-Akt (#13038), Bax (#14796), Bcl-2 (#2764), and cleaved-caspase-3 (#9664) were obtained from Cell Signaling Technology. Antibodies against MBP (ab40390), NF200 (ab4680), Nrf2 (ab62352), NQO1 (ab34173), MPZ (ab31851), and Akt (ab179463), Histone H3 (ab176842) and the PI3K/Akt inhibitor LY294002 (ab120243), were purchased from Abcam. Antibodies against GAPDH (10494-1-AP), HO-1 (10701-1-AP), SOD2 (24217-1-AP), and PCNA (10205-2-AP) were purchased from Proteintech. An antibody against S100 (sc-53438) was obtained from Santa Cruz Biotechnology.

### Animals

Male SD rats (200∼220 g) were purchased from the Laboratory Animal Center of Fujian Medical University (Fujian, China). A temperature of 23 ± 2 °C, a humidity of 35-60%, and a 12:12 h light-dark cycle were applied as standardized laboratory conditions for housing all rats. Meanwhile, they were provided with food and water and were habituated to these conditions for at least 7 days before the experiment. The use of animals in this study was approved by the Animal Experimentation Ethics Committee of Wenzhou Medical University, Wenzhou, China. The living conditions and experimental procedures were conducted in accordance with the National Institutes of Health Guideline concerning the Care and Use of Laboratory Animals.

### PNI Model and Drug Injection

The procedure for generating the animal model was described previously (Li et al., 2017b). In brief, each animal was anaesthetized by an intraperitoneal injection of 10% chloral hydrate (3.5 ml/kg). An incision was made in the skin to expose the right sciatic nerve. Then, this exposed nerve was crushed with two vascular clips (Oscar, China). The vascular clips clamped the sciatic nerve 7 mm proximal from the sciatic notch at the two ends with 30 g of force for 2 min. Thereafter, the incised skin was sutured with a 4-0 nonabsorbable suture.

Following surgery, all the animals were randomly allocated to four groups (n = 10 for each): the sham group, the PNI group, the FGF10 group, and the FGF10+LY294002 group. The sham group received the same surgical procedure to expose the sciatic nerve but did not undergo compression injury. For the FGF10 group, each rat was injected intramuscularly with 5 μg of FGF10 solution (25 μg/ml) once daily for 28 consecutive days. For the FGF10 + LY294002 group, after the injection of FGF10 solution, each rat was also intravenously injected with LY294002 (a PI3K inhibitor, 0.3 mg/kg/day) (Chen et al., 2017). The rats in the sham and PNI groups were only injected with the same volume of saline solution. After 28 days, all rats were sacrificed to harvest the sciatic nerve for pathology index analysis.

### Von Frey Test and Walking Track Analysis

Rats were habituated to an elevated metal mesh floor for at least 1 h before their responses to mechanical stimulation were tested with von Frey filaments (NC12775; North Coast Medical Inc, CA, USA). von Frey filaments with forces ranging from 2–180 g were applied in ascending order to the third and fourth toes of the plantar surface of the hind paw until they bent. The filaments were repeated five times, and the results of paw withdrawal were recorded.

The gait of the rats along a 100 × 10 × 15 cm corridor, the bottom of which was covered with a 90 × 10 cm piece of white paper, was analyzed. Red ink was painted on the hind paws of the rats. The sciatic functional index (SFI) was calculated based on the colored footprints according to the following formula by Bain et al. (1989): SFI = −38. 3 × (EPL- NPL)/NPL + 109.5 × (ETS -NTS)/NTS + 13.3 × (EIT - NIT)/NIT-8.8, where E is the right hind limb; N is the left hind limb; PL is the longitudinal distance of the longest footprint; IT is the distance between the second and fourth toes; and TS is the distance between the first and fifth toes. An index of approximately 0 reflects normal function, whereas an index of −100 indicates complete impairment. Two observers who were unaware of the experimental procedures performed the test from day 1 to day 28 following the surgical procedures.

### RSC96 Culture and Treatment

The RSC96 SC line was obtained from ScienCell Research Laboratories. The cells were cultured in Dulbecco’s modified Eagle medium (DMEM) with 10% fetal bovine serum (FBS, Gibco, USA) and 1% penicillin/streptomycin solution (P/S) in a humidified incubator (37°C, 5% CO_2_). After two passages, the cells were seeded in 96-well plates (5 × 10^3^ cells/well), and various concentrations of FGF10 (0.043, 0.43, 4.3, 43 nm) were added for 2 h. Then, the medium was supplemented with 100-μm H_2_O_2_ for another 2 h. To further evaluate the effect of PI3K/Akt activation on oxidative injury, cells were pretreated with the PI3K inhibitor LY294002 (20 μm) (Wang et al., 2012) for 2 h before the addition of FGF10.

To evaluate cell survival, a cell counting kit (CCK-8, Beyotime Institute of Biotechnology, China) was used. The test was performed according to the manufacturer’s instructions. In brief, 10 μl of CCK-8 solution were added to each well, and the cells were incubated for 2 h at 37°C. The optical density was measured at 450 nm using a microplate reader (Thermo Fisher Scientific, Waltham, MA). All experiments were repeated at least three times.

### Assays of Intracellular ROS Generation

Intracellular ROS generation was measured by an ROS Assay Kit (DCFH-DA, S0033, Beyotime, China). SCs were plated in 6-well plates at a density of 1×10^5^ cells/ml for 24 h. After treatment with H_2_O_2_ with/without FGF10 plus LY294002, 10 μm 2′, 7′-dichlorodihydrofluorescein diacetate (DCFH-DA) were added to the culture medium for 20 min. Subsequently, the fluorescence of the cells in five randomly fields for each group was imaged with a Nikon ECLIPSE 80i microscope (Nikon, Japan). ImageJ software was used for quantitative analysis.

To further analyze the change of ROS, the DCF fluorescence was detected by a fluorescence microplate reader (Genios, TECAN) at 485-nm excitation and 53-nm emission.

### Hematoxylin-Eosin (He) Staining

The sciatic nerve tissues were harvested following the established methods and embedded in paraffin (Li et al., 2017b). The nerves were fixed in 4% paraformaldehyde in 0.1 M phosphate buffer overnight and embedded in paraffin the next day. The longitudinal sections were cut into 5-μm thick sections for HE staining; the sections were dyed with hematoxylin for 5 min and with eosin for 10 min. The images were captured using a Nikon ECLIPSE 80i microscope (Nikon, Japan).

### Immunoblotting

Immunoblotting analysis of sciatic nerves and cell extracts were performed as described previously (Zhang et al., 2014; Li et al., 2018b). Briefly, cells and sciatic nerves were lysed using RIPA with protease (Boster, AR0101/AR0103) and phosphatase inhibitors (Applygen, P1260). The lysate was centrifuged at 12 000 g for 20 min. For PCNA detection, the sciatic nerve segments was mechanically homogenized. Then, cytosol and nuclear proteins were extracted using the nuclear and cytoplasmic protein extraction kit (Beyotime Biotechnology, Wuhan, China) and centrifuged at 5000 r.p.m. for 10 min at 4°C to extract the nuclear components (Li et al., 2015a). The protein concentration was determined by bicinchoninic acid (BCA) reagents (Thermo Fisher Scientific, Rockford, IL, USA). Sixty micrograms of protein was separated by 8-12% SDS-PAGE and transferred onto PVDF membranes (Bio-Rad, Hercules, CA, USA). After blocking with 5% nonfat milk, the membranes were incubated with primary antibodies overnight at 4°C. The antibodies included *p*-Akt (1:1000), Akt (1:1000), Nrf2 (1:1000), NQO1 (1:1000), HO-1 (1:1000), SOD2 (1:2000), MPZ (1:1000), S100 (1:200), PCNA (1:1000), Bcl-2 (1:1000), Bax (1:1000), Histone H3 (1:1000) and GAPDH (1:10000). The next day, the membranes were incubated with horseradish peroxidase-conjugated secondary antibodies for 1 h. The visualization of signals and band intensity were analyzed by chemiluminescence using a gel imaging system (Bio-Rad Laboratories, Hercules, CA, USA). Three independent samples were analyzed.

### Immunofluorescence Staining

For animal tissues, 5-μm thick longitudinal sections were washed in PBS three times after being embedded in optimum cutting temperature (OCT) compound. For cells, SCs were fixed in 4% paraformaldehyde in PBS for 20 min. Then, the tissue or cell samples were blocked in 5% bovine serum albumin (BSA) containing 0.1% Triton X-100 for 30 min. After incubation with primary antibodies overnight at 4°C, the slides were incubated with a fluorochrome-labeled secondary antibody for 1 h. The primary antibodies included NF200 (1:10000), MBP (1:1000), and Nrf2 (1:1000). The nuclei were stained with DAPI for 7 min. Image acquisition was performed with a Nikon ECLIPSE Ti microscope (Nikon, Tokyo, Japan).

### Statistical Analysis

All data are expressed as the mean ± SEM. Analysis of statistical significance was performed by one-way ANOVA with Tukey’s posttest with GraphPad Prism (GraphPad Software version 6.0, La Jolla, CA, USA). *P*-values less than 0.05 were considered statistically significant.

## Results

### Fgf10 Promotes Functional Recovery

**Figure 1:**
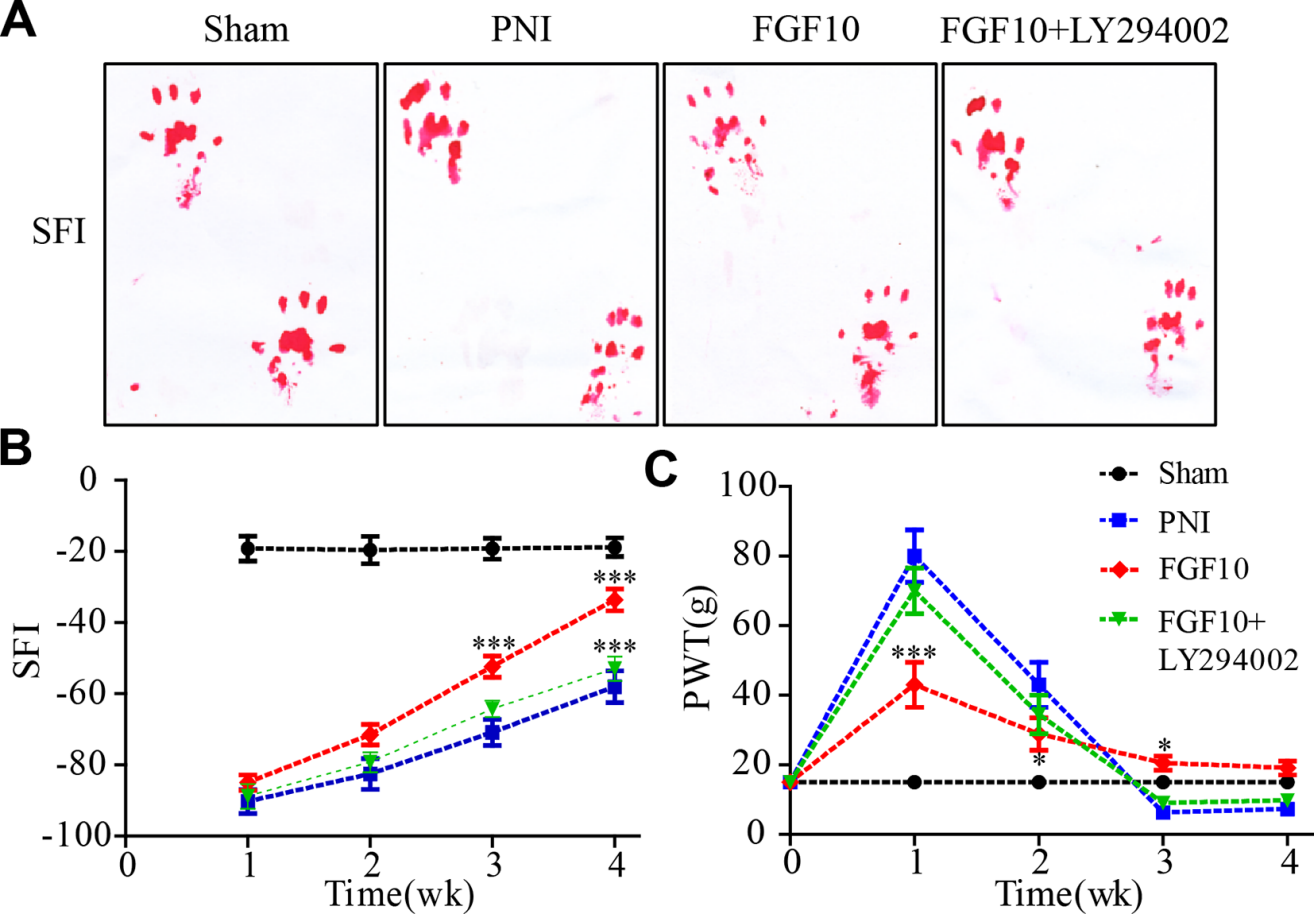
Fibroblast growth factor 10 (FGF10) enhances motor and sensory functional recovery after peripheral nerve injury (PNI). **(A)** Photographs of rat footprints 4 weeks after sciatic nerve crush. **(B)** Statistical analysis of the sciatic functional index (SFI) at the indicated times postoperatively. **(C)** Paw mechanical withdrawal thresholds were measured at predetermined time points. FGF10 group vs PNI group: *P < 0.05, ***P < 0.001. FGF10 group vs FGF10+ LY294002 group: ***P < 0.001. All data represent the mean values ± SEM; n = 8 in each group.

To assess whether the injection of FGF10 increase sensory and motor recovery, walking track analysis and paw withdrawal thresholds were evaluated every week for 4 weeks following surgery. The walking tracks indicated no significant difference among the three groups in the first 2 weeks after surgery. However, the SFI value of the FGF10-treated group began to increase and was significantly greater than that of the PNI group at weeks 3 and 4 (**Figures 1A, B**, ^***^ *P* < 0.001). Nevertheless, compared with the group that received FGF10 only, the group that received FGF10 and LY294002 co-administration exhibited motor functional recovery by week 4 (**Figures 1A, B**, ^***^ *P* < 0.001).

To evaluate sensory functional recovery to mechanical stimuli, all animals from the four groups were subjected to the von Frey test. As illustrated in **Figure 1C**, the sensory recovery of all groups was rather poor 1 week after crush injury, but recovery in the FGF10 group was better than that in the PNI group (^***^ *P *< 0.001). The mechanical thresholds gradually decreased in the subsequent weeks. Although the results seemed to show that sensory recovery in the FGF10 group was inferior to that in the PNI group and FGF10 + LY294002 group, the withdrawal threshold in the two latter groups was even lower than that in the control group, suggesting that the PNI rats treated with/without FGF10+LY294002 exhibited hyperalgesia and that FGF10 treatment rescued PNI-induced hyperalgesia at a later stage of nerve recovery. Taken together, these results suggest that FGF10 continuously enhances the recovery of locomotor and sensory function in acute PNI.

### Fgf10 Facilitates Nerve Regeneration

**Figure 2:**
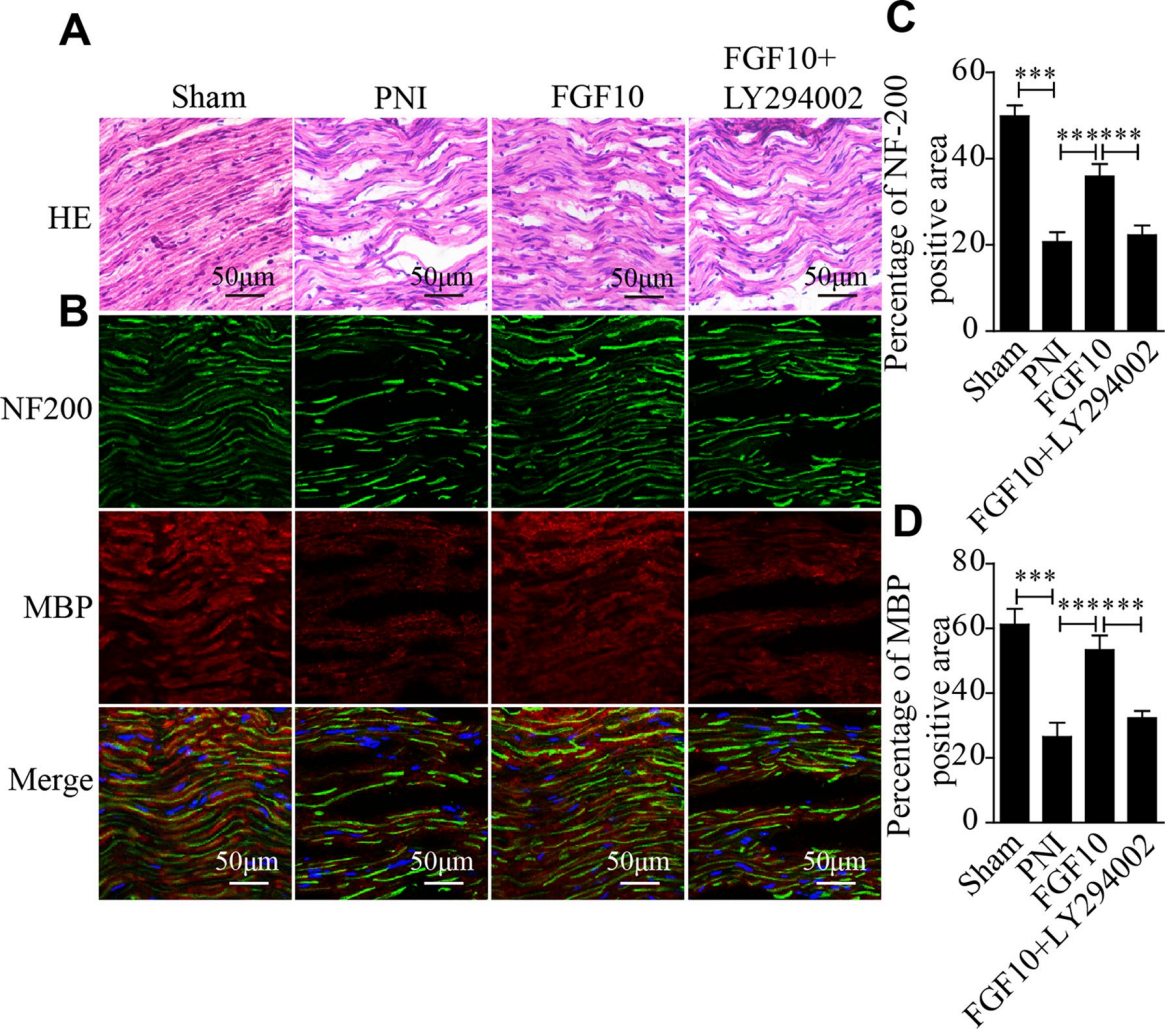
Fibroblast growth factor 10 (FGF10) promotes axonal regeneration, as determined by histological evaluation. **(A)** Longitudinal sections of regenerated nerves from each group were stained with Hematoxylin-Eosin (HE) 28 days postinjury. Scale bars = 50 μm. **(B)** Immunofluorescence staining for myelin basic protein (MBP) (red) and NF-200 (green) in longitudinal sections. Scale bars = 50 μm. **(C, D)** Quantitative analysis of the fluorescence intensity of MBP and NF-200 28 days following injury. The data are presented as the mean ± SEM, n = 5. Sham group vs PNI group: ***P < 0.001. FGF10 group vs peripheral nerve injury (PNI) group: ***P < 0.001. FGF10 group vs FGF10+LY294002 group: ***P < 0.001.

The histological recovery of the injured nerve in each group was evaluated by HE staining and coimmunostaining for neurofilament 200 (NF-200; represents axonal growth) and MBP (a marker of myelination). HE staining revealed that the axonal fibers in the PNI group were scarce and irregular, while the nerve fibers in the FGF10-treated group appeared remarkably regenerated and regular (**Figure 2A**). Double immunofluorescence staining for MBP and NF200 showed that the density of regenerated axons and myelin in the FGF10 group was significantly higher than that in the PNI group and that the degree of regeneration was enough to induce a morphology similar to that of normal nerves (**Figures 2B–D**). In contrast, the immunoreactivity of NF200 and MBP was greatly attenuated after the coadministration of LY294002 and FGF10.

### Fgf10 Increases Functional Protein Secretion

**Figure 3:**
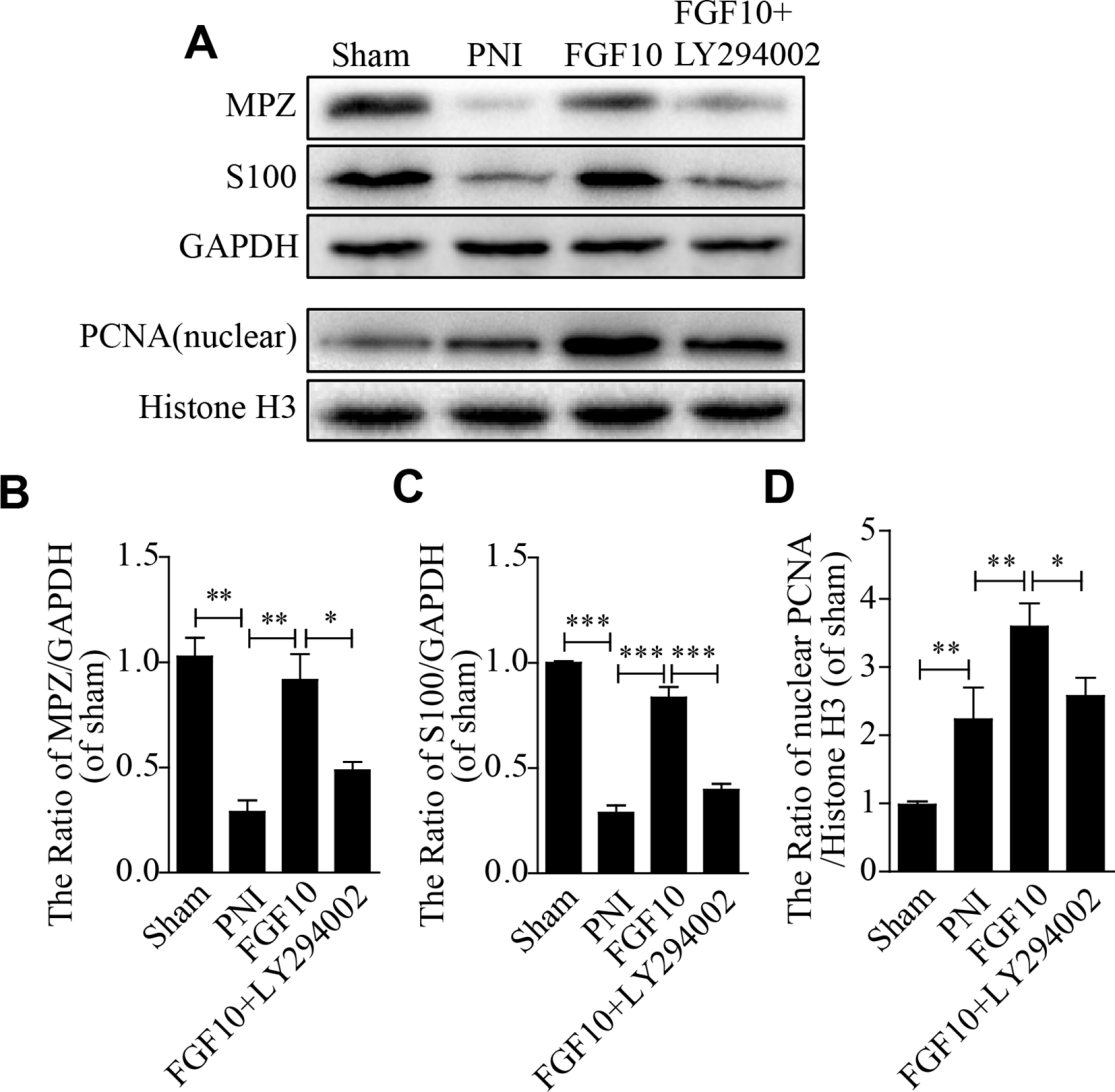
Fibroblast growth factor 10 (FGF10) enhances the expression of functional proteins after sciatic nerve injury. **(A**–**D)** Representative immunoblotting images of myelin basic zero (MPZ), S100, and proliferating cell nuclear antigen (PCNA) expression and the quantification of protein levels in sciatic nerve lesions 28 days postinjury. The data are presented as the mean ± SEM, n = 3. Sham group vs peripheral nerve injury (PNI) group: **P < 0.01, ***P < 0.001. FGF10 group vs PNI group: **P < 0.01, ***P < 0.001. FGF10 group vs FGF10+LY294002 group: *P < 0.05, **P < 0.01, ***P < 0.001.

S100 is a SC marker that regulates cellular metabolism, motility and proliferation. Myelin protein zero (MPZ; also called P0) is a major extrinsic membrane protein of myelin in the PNS. MPZ function includes forming myelin and maintaining compact myelin morphology. PCNA is a nucleoprotein that is a marker of cell proliferation. The expression of these proteins was quantified using western blotting analysis. As shown in **Figure 3A**, the protein expression of S100, MPZ and PCNA in the FGF10 group was significantly increased compared with that in the PNI group, while this effect was reversed by the injection of LY294002. Quantitative analysis also showed the same trend (**Figures 3B–D**). These data reveal that the beneficial effect of FGF10 is able to upregulate the functional expression of these proteins and that this effect further contributes to SC remyelination and axonal regeneration.

### FGF10 Inhibits the Excessive Expression of Oxidative Stress- and Apoptosis-Related Proteins by Activating PI3k/Akt Signaling

**Figure 4:**
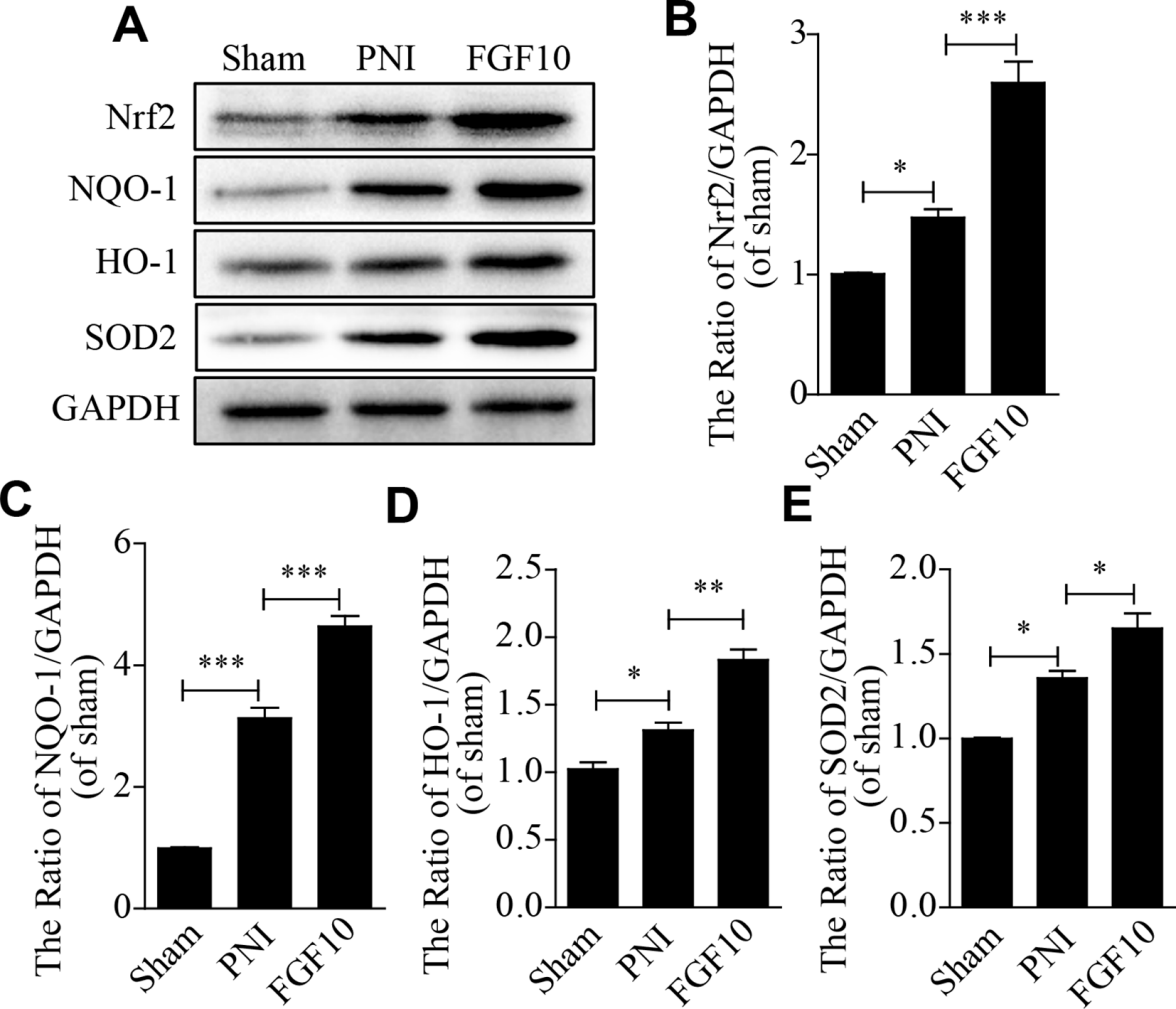
Fibroblast growth factor 10 (FGF10) suppresses oxidative stress after peripheral nerve injury (PNI). **(A**-**E)** western blotting analysis showed the expression of Nrf2, NQO1, HO-1, and SOD2 after treatment with FGF10. Data are presented as the mean ± SEM, n = 3. Sham group vs PNI group: *P < 0.05, ***P < 0.001. FGF10 group vs PNI group: *P < 0.05, **P < 0.01, ***P < 0.001.

To test whether FGF10 treatment inhibits PNI-induced oxidative stress in the sciatic nerve, the expression of oxidative stress-related proteins, including Nrf2, NQO1, SOD2 and HO-1, was detected by western blotting. The levels of these oxidative stress-associated molecules were slightly increased by PNI. FGF10 treatment further increased the production of these antioxidant proteins to a large degree **(** **Figures 4A–E**).

**Figure 5:**
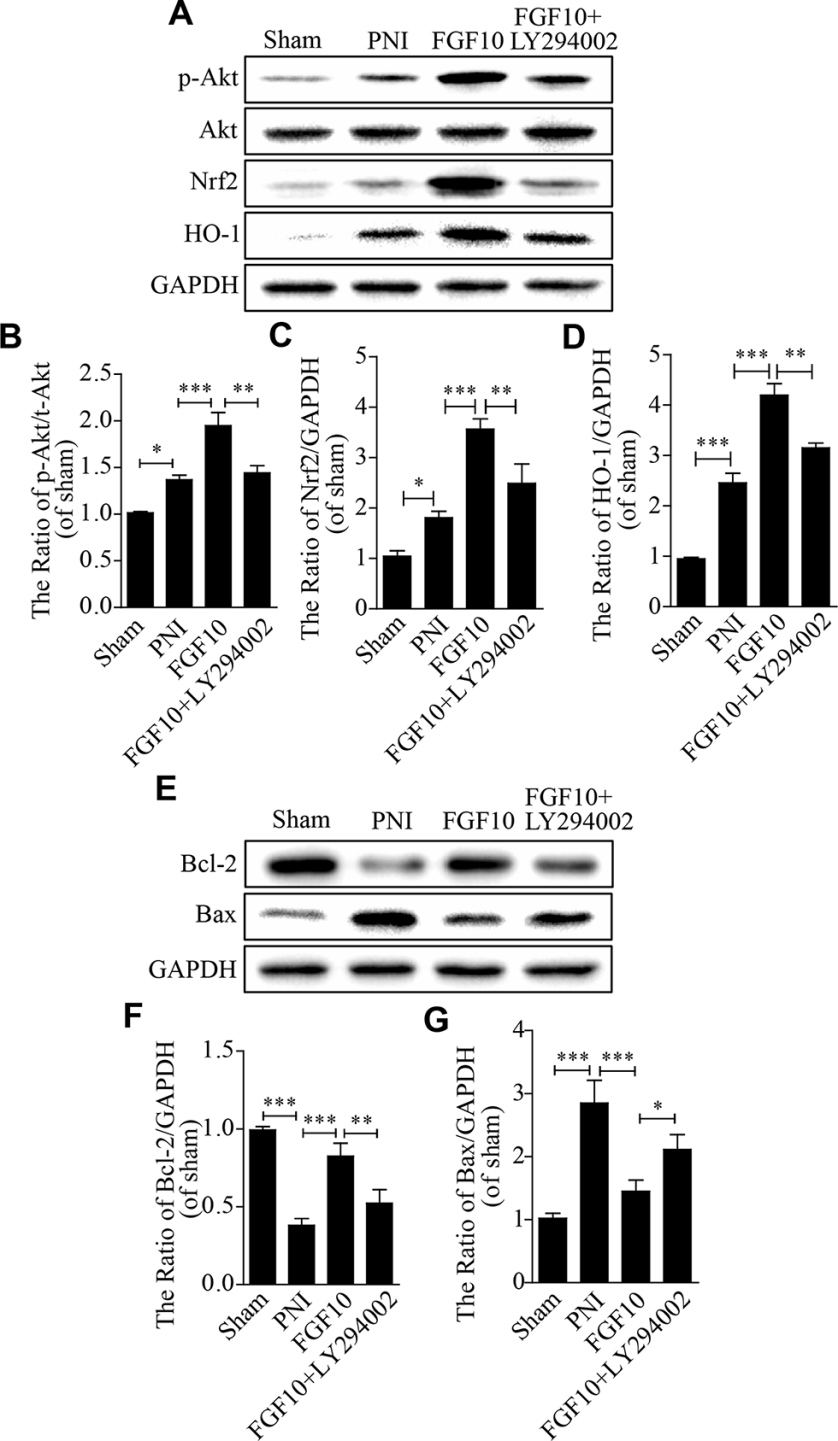
The antioxidative and antiapoptotic functions of fibroblast growth factor 10 (FGF10) are mediated by the activation of the PI3K/Akt pathway *in vivo*. **(A**–**D)** Representative western blots and the quantification of *p*-Akt, Akt, Nrf2, and HO-1 expression in each group. **(E**–**G)** Western blotting and the quantification of Bcl-2 and Bax expression 28 days after injury. The data are presented as the mean ± SEM, n = 3. Sham group vs peripheral nerve injury (PNI) group: *P < 0.05, ***P < 0.001. FGF10 group vs PNI group: ***P < 0.001. FGF10 group vs FGF10+LY294002 group: *P < 0.05, **P < 0.01.

Previous studies have demonstrated that the recovery of neurological deficits is closely related to the activation of the PI3k/Akt pathway (Wu et al., 2016; Sang et al., 2018). Inspired by this fact, we measured the *p*-Akt and Akt levels using western blotting. Here, we found that the levels of Akt phosphorylation were slightly increased after PNI, and these levels were further upregulated after PNI rats received FGF10 treatment, which was also confirmed by the *p*-Akt/Akt ratio. However, LY294002 dramatically inhibited this effect on the expression of these proteins (**Figures 5A, B**). Nrf2 and HO-1 showed nearly the same trend (**Figures 5A, C, D**).

We also detected the expression of apoptosis-related proteins (including Bax and Bcl-2) to evaluate whether the anti-oxidative capability of FGF10 helps to decrease cell apoptosis following PNI. Western blotting showed that the level of the pro-apoptotic protein Bax was down-regulated and that the level of the anti-apoptotic protein Bcl-2 was up-regulated in the FGF10 group when compared to the PNI group (**Figures 5E–G**). However, the administration of LY294002 partially abolished the effects of FGF10. These results confirm that the antioxidant and anti-apoptotic properties of FGF10 may be involved in the activation of PI3k/Akt signaling.

### Fgf10 Reduces Sc Apoptosis *in Vitro*

**Figure 6:**
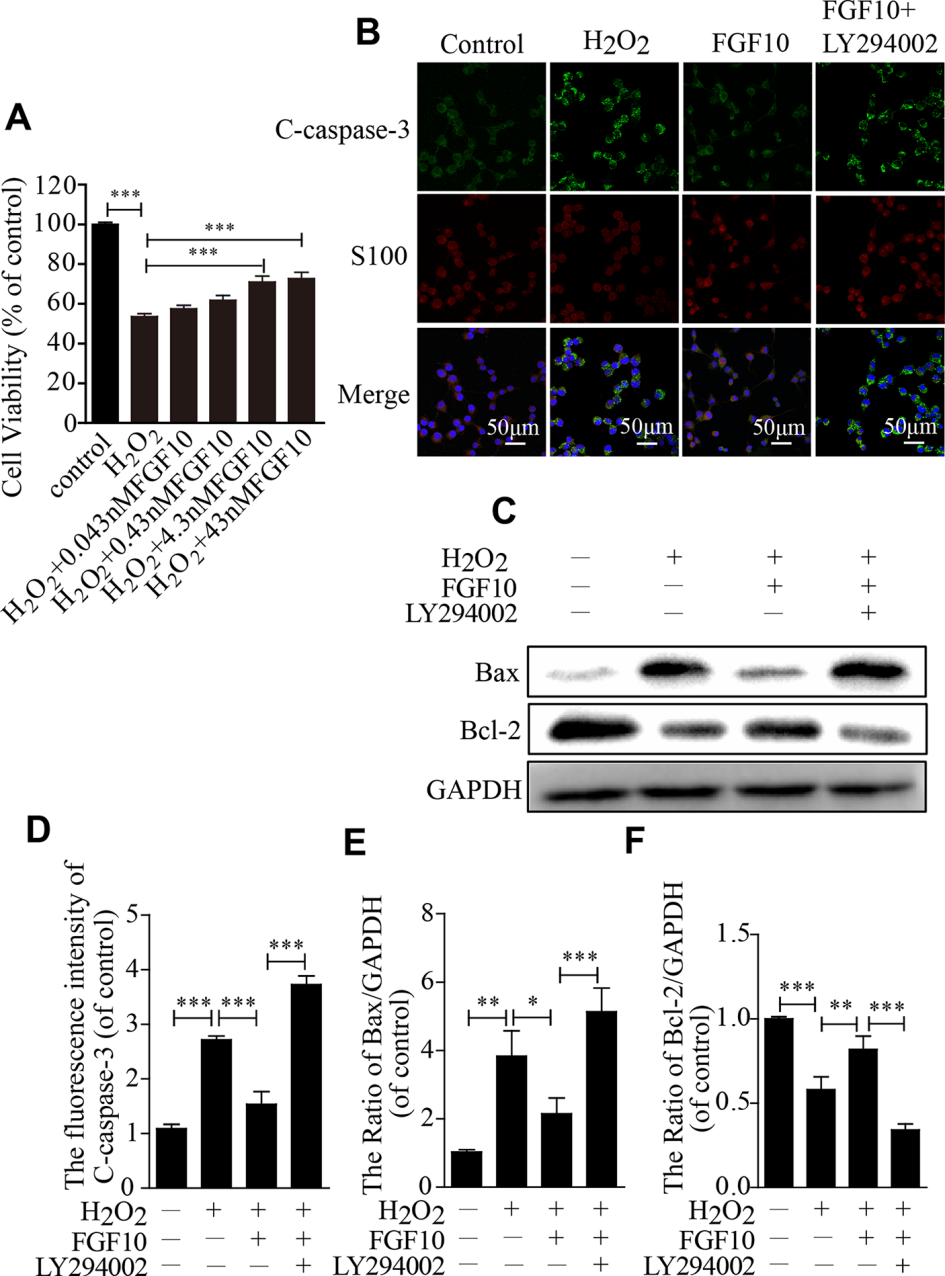
Fibroblast growth factor 10 (FGF10) inhibits Schwann cell (SC) apoptosis *in vitro*. **(A)** H_2_O_2_-induced cell survival was evaluated by the CCK-8 assay. **(B)** Immunofluorescence staining results of cleaved-caspase-3 (green) and S100 (red) in each group. Scale bar = 50 μm. **(C)** The protein levels of Bax and Bcl-2 were detected by western blotting. **(D)** Statistical analysis of the cleaved-caspase-3 intensity in each group. **(E**, **F)** The quantification of Bax and Bcl-2 expression was assessed *via* a gel imaging system. All of these data represent the means ± SEM, n = 3. Control group vs H_2_O_2_ group: **P < 0.01, ***P < 0.001. FGF10 group vs H_2_O_2_ group: *P < 0.05, **P < 0.01, ***P < 0.001. FGF10 group vs H_2_O_2_+LY294002 group: ***P < 0.001.

To further confirm the protective effect of FGF10, SCs were exposed to 100-μm H_2_O_2_ alone or in combination with various concentrations of FGF10. The CCK-8 results showed that cell viability increased as the FGF10 concentration increased and that 4.3-nm FGF10 was the most effective concentration (**Figure 6A**). Therefore, this concentration of FGF10 was chosen for subsequent experiments. Double immunostaining for cleaved-caspase-3 and S100 showed that FGF10 significantly reduced the cleaved-caspase-3 signal intensity, and LY294002 partially reversed this effect (**Figures 6B, D**). Consistent with the immunofluorescence, SC apoptosis in all four treatment groups was further confirmed *via* detecting Bax and Bcl-2 expression levels by western blotting (**Figures 6C, E, F**). These data suggest that FGF10 maintains SC bioactivity under H_2_O_2_ exposure.

### Fgf10 Alleviates Oxidative Injury Through Pi3k/Akt Signaling *in Vitro*

**Figure 7:**
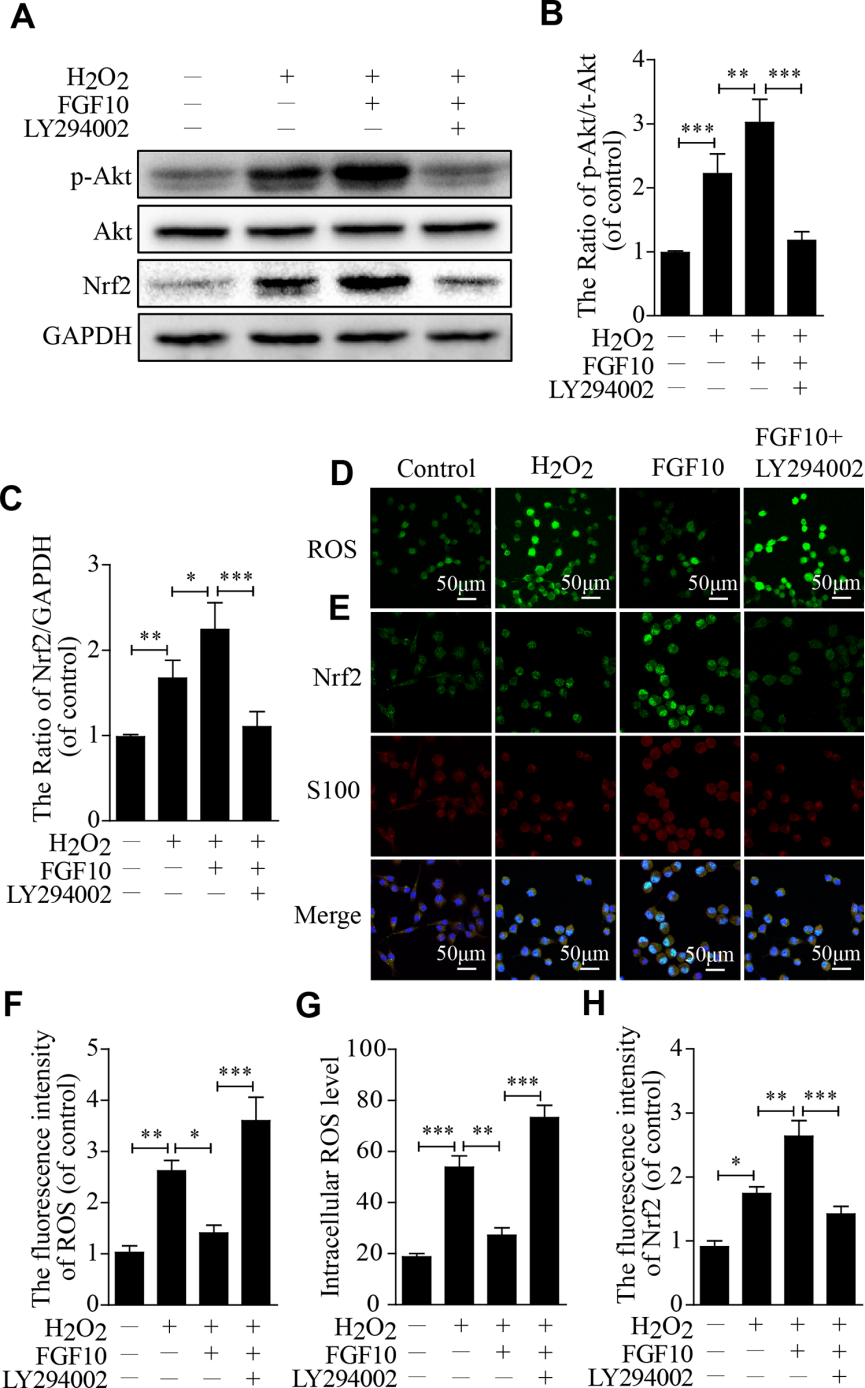
Fibroblast growth factor 10 (FGF10) has antioxidant effects through the activation of the PI3K/Akt pathway *in vitro*. **(A**–**C)** Then, quantification of *p*-Akt, Akt, and Nrf2 expression was determined by western blotting in Schwann cells (SCs). **(D)** Immunofluorescence staining of reactive oxygen species (ROS) labeled by dichlorodihydrofluorescein diacetate (DCFH-DA) in SCs. **(E)** Double immunofluorescence of Nrf2 (green) and S100 (red) in each group. Scale bar = 50 μm. **(F**–**H)** Statistical analysis of the ROS and Nrf2 levels in each group. These data represent the means ± SEM, n = 3. Control group vs H_2_O_2_ group: *P < 0.05, **P < 0.01, ***P < 0.001. FGF10 group vs H_2_O_2_ group: *P < 0.05, **P < 0.01. FGF10 group vs H_2_O_2_+LY294002 group: ***P < 0.001.

To investigate the molecular mechanism by which FGF10 protects SCs against H_2_O_2_-induced apoptosis *in vitro*, we first detected the *p*-Akt and Akt levels using western blotting, the change in the *p*-Akt/Akt ratio was slightly increased in the H_2_O_2_ group and was further increased in the FGF10-treated group. This upregulation was reversed by LY294002 treatment. Nrf2 showed nearly the same trend (**Figures 7A–C**).

The change in ROS levels in each group since ROS represents the core component of the biology of oxidative stress. Compared with that in the H_2_O_2_-treated group, the fluorescence intensity was lower in the FGF10 group but was significantly higher in the FGF10+LY294002 group (**Figures 7D, F**). The intracellular ROS level was as similar change as the fluorescence intensity (**Figure 7G**). However, Nrf2 expression was slightly increased in the H_2_O_2_ group, which further increased in the FGF10-treated group. This upregulation was markedly reduced after the addition of LY294002 (**Figures 7E, H**).These results reveal that FGF10 may have the potential to enhance antioxidant ability *via* activating PI3K/Akt signaling.

## Discussion

Nerve recovery in the PNS is a complex process that triggers a sequence of events, including axonal regeneration, SC proliferation and migration, macrophage infiltration, and angiopoiesis, within the lesion area. During this process, the FGF family is particularly important for directing cellular activity and tissue remodeling. FGFs participate in SC dedifferentiation, proliferation, and remyelination during axonal regrowth (Chen et al., 2007; Jessen et al., 2015). FGF secretion is essential for maintaining neuronal survival after PNI. Furthermore, numerous studies have reported that FGFs, such as basic FGF (bFGF) and nerve growth factor (NGF), are essential for supporting neurite outgrowth and functional recovery during nerve regeneration (Grothe et al., 2006; Sun et al., 2009; Chen et al., 2010; Takagi et al., 2012; Li et al., 2017a). However, there is no knowledge of whether FGF10 promotes nerve regeneration after PNI.

In this article, we reported a new role for FGF10 in improving sensory and motor functional recovery, enhancing axonal regrowth and remyelination, and increasing the expression of functional proteins after traumatic PNI. Moreover, FGF10-induced neuroprotection and neuranagenesis is associated with attenuating the acute activation of oxidative stress and apoptosis in SCs, which is likely regulated by the activation of PI3K/Akt signaling. These findings indicate that FGF10 may be regarded as a potential therapeutic agent for peripheral nerve reconstruction after injury.

FGF10, a typical paracrine growth factor, is also essential for tissue development and regenerative medicine through specifically binding to the epithelial receptor FGFR2b (Zhang et al., 2006). The exogenous supplementation of FGF10 has been shown to prevent the formation and development of numerous diseases, including wound healing deficits, cardiovascular diseases, metabolism syndrome, and acute kidney injury (Konishi et al., 2006; Rochais et al., 2014; Li et al., 2017c; Tan et al., 2018). Previous studies have shown that FGF10 protects neurons against inflammation-induced apoptosis during SCI through the activation of FGFR2/PI3K/Akt signaling (Chen et al., 2017). However, the effect of FGF10 in acute PNI *in vivo* is unknown. In the present study, we reported that FGF10 is sufficient to continuously enhance motor and sensor recovery, improve nerve morphological recovery and reconstruction, and reduce SC apoptosis. These advantages suggest that FGF10 may be a potential therapeutic agent for peripheral nerve repair.

Oxidative stress is a redox-reactive imbalance in which the generation of oxygen-free radicals and ROS is far greater than the formation of antioxidative entities. During PNI, excessive oxidative stress activation is closely linked to SC apoptosis and axonal atrophy, which is unfavorable for nerve repair (Mirzakhani et al., 2018; Ullah et al., 2018; Lu et al., 2019). Numerous lines of evidence have implicated PI3K/Akt signaling in modifying oxidative stress (Pan et al., 2017; Liu et al., 2018; Gong et al., 2018; Li et al., 2018a). Hyperglycemia-induced oxidative stress can be negatively regulated by PI3K/Akt signaling (Pan et al., 2017). Furthermore, previous research has shown that the neuroprotective effect of fibroblast growth factor 21 (FGF21) in promoting neuronal survival and neurofunctional recovery after brain injury is mediated by the activation of PI3K/Akt signaling (Lu et al., 2019). In addition, the PI3K/Akt signaling pathway also participates in neuronal differentiation, survival and synaptic function (Li et al., 2006; Ketschek and Gallo, 2010; Fu et al., 2014). Given the important role of PI3K/Akt signaling in promoting cell survival and resisting cellular stress, we hypothesize that PI3K/Akt signaling may act as an important antioxidant mechanism for regulating FGF10-induced neuroprotection and neuranagenesis following PNI.

Here, we found that the levels of stress-related proteins, including Nrf2, NQO1, SOD2, and HO-1, and the ratio of *p*-Akt/Akt increased markedly; meanwhile, ROS generation and the level of apoptosis were significantly decreased after the administration of FGF10. Suppressing Akt phosphorylation with LY294002 partially reversed these therapeutic effects. This result might be explained that the nerve tissue had the capable of synthetizing and secreting certain amounts of antioxidant enzymes to resist PNI-induced oxidative damage, although this capability was not satisfied the demand of cellular antioxidant defense (Ornitz and Itoh, 2015; Yu et al., 2017). After administration of FGF10 to the PNI rats, the interaction between FGF10-FGFR pairs activated the downstream of signal transduction pathways, such as PI3K/Akt, to dramatically stimulate the expression of antioxidant proteins which further enhanced antioxidative capability and, therefore, promoting SCs proliferation and nerve regeneration. Consequently, FGF10-induced nerve regeneration and functional recovery may occur through PI3K/Akt signaling-mediated antioxidant enhancement.

Previous studies reported that oxidative-induced SCs apoptosis is a critical mechanism of neurodegenerative diseases (Purves et al., 2001). In mammalian cells, Bax and Bcl-2 are involved in the regulation of apoptosis. Among them, Bax belongs to proapoptotic gene, whereas Bcl-2 is antiapoptotic gene. Abnormal oxidative stress activity triggers a decreased expression of Bcl-2 and an increased expression of Bax, which severely influences cell survival and induce cell apoptosis (Jezek and Plecitá-Hlavatá, 2009). Moreover, the excessive ROS-induced apoptosis after PNI is closely associated with the ratio of activity of Bax/Bcl-2 (Zhang et al., 2016). In our study, we observed that the downregulation of Bcl-2 and the upregulation of Bax after PNI were reversed after treatment of FGF10, while this effect was partially abolished when combination of FGF10 and LY294002 together. This result indicates that ROS-induced apoptosis after PNI is probably regulated by PI3K/Akt signaling.

In conclusion, our data indicate that the administration of FGF10 can be effective in facilitating SC proliferation, axonal regeneration, and functional recovery after sciatic nerve injury. In addition, the neuroprotective effect of FGF10 treatment is likely associated with suppressing excessive oxidative stress-induced cell apoptosis *via* activating PI3K/Akt signaling. Thus, our research may provide an alternative therapeutic strategy for utilizing FGF10 for treating acute traumatic PNI.

## Data Availability Statement

All datasets generated for this study are included in the manuscript/supplementary files.

## Ethics Statement

The animal study was reviewed and approved by Male SD rats (200∼220g) were purchased from Laboratory Animal Center of Fujian Medical University (Fujian, China). The temperature between 23 ± 2°C, humidity between 35 and 60%, and a light-dark cycles with the ratio of 12:12h were applied as the standardized laboratory conditions for housing all the rats. Meanwhile, they were provided with food and water, and adjusted to this condition for at least 7 days before experiment. The animals used in this study were approved by the Animal Experimentation Ethics Committee of Wenzhou Medical University, Wenzhou, China. The living condition and experimental procedures were conducted in accordance to the National Institutes of Health Guideline concerning the Care and Use of Laboratory Animals.

## Author Contributions

XJ and WJ conceived and designed the research. DL, LD, and WB performed the experiments. DL and LR performed the statistical analysis and wrote the paper. LY, LF, YF, NX, JY, WY, YS, LG, and LX provided assistance with the experiments. All authors discussed the drafting of the manuscript.

## Funding

This work was supported by grants from the National Natural Science Foundation of China (81802238,81972150, 81722028), Zhejiang Provincial Natural Science Foundation of China (LR18H50001) and the Project of Wenzhou Science and Technology Bureau(2018Y0498,2015Y0416, 2015Y0235).

## Conflict of Interest

The authors declare that the research was conducted in the absence of any commercial or financial relationships that could be construed as a potential conflict of interest.

The handling editor is currently organizing a Research Topic with one of the authors, XL, and confirms the absence of any other collaboration.

## Abbreviations

bFGF, basic fibroblast growth factor; BSA, bovine serum albumin; CCK-8, cell counting kit; CNS, central nervous system; DCFH-DA, 2′, 7′-dichlorodihydrofluorescein diacetate; DMEM, Dulbecco’s modified Eagle medium; FBS, fetal bovine serum; FGFs, fibroblast growth factors; FGF10, fibroblast growth factor 10; FGF21, fibroblast growth factor 21; HE, hematoxylin and eosin; NGF, nerve growth factor; OCT, optimum cutting temperature; PNI, peripheral nerve injury; PNS, peripheral nervous system; PWT, paw withdrawal threshold; P/S, penicillin/streptomycin; ROS, reactive oxygen species; SCI, spinal cord injury; SCs, Schwann cells; SFI, sciatic function index.
